# Regulatory B cells preferentially accumulate in tumor-draining lymph nodes and promote tumor growth

**DOI:** 10.1038/srep12255

**Published:** 2015-07-20

**Authors:** Sheila N. Ganti, Tina C. Albershardt, Brian M. Iritani, Alanna Ruddell

**Affiliations:** 1 Department of Comparative Medicine, University of Washington, Seattle, WA, USA; 2 Fred Hutchinson Cancer Research Center, Seattle, WA, USA

## Abstract

Our previous studies found that B16-F10 melanoma growth in the rear footpad of immunocompetent mice induces marked B cell accumulation within tumor-draining popliteal lymph nodes (TDLN). This B cell accumulation drives TDLN remodeling that precedes and promotes metastasis, indicating a tumor-promoting role for TDLN B cells. Here we show that phenotypic characterization of lymphocytes in mice bearing B16-F10 melanomas identifies preferential accumulation of T2-MZP B cells in the TDLN. Comparison of non-draining LNs and spleens of tumor-bearing mice with LNs and spleens from naïve mice determined that this pattern of B cell accumulation was restricted to the TDLN. B cell-deficient and immunocompetent mice reconstituted with T2-MZP B cells but not with other B cell subsets displayed accelerated tumor growth, demonstrating that T2-MZP B cells possess regulatory activity in tumor-bearing mice. Unlike splenic regulatory B cells, however, these TDLN B cells did not exhibit increased IL-10 production, nor did they promote Treg generation in the TDLN. These findings demonstrate that tumors initially signal via the lymphatic drainage to stimulate the preferential accumulation of T2-MZP regulatory B cells. This local response may be an early and critical step in generating an immunosuppressive environment to permit tumor growth and metastasis.

Metastasis to lymph nodes (LNs) is the most important factor in predicting cancer spread to distant organs in many types of tumors, including breast cancer and melanoma^1^. However, the mechanism by which the tumor modifies the draining LN to facilitate metastasis is poorly understood. Subcutaneous implantation of B16-F10 melanoma cells in the rear footpad of mice induces hypertrophy of the draining popliteal LN, which precedes and predicts melanoma metastasis^2^,^3^. The tumor-draining LN (TDLN) demonstrates increased lymphocyte cellularity with an 8- and 3-fold accumulation of B and T cells, respectively. This lymphocyte accumulation is associated with extensive growth of the lymphatic sinuses (lymphangiogenesis) and a 20-fold increase in lymph flow through the TDLN compared to the non-TDLN (NTDLN)^2^. Moreover, enforced B cell accumulation in LNs of preneoplastic Eμ-*c-myc* mice drives LN lymphangiogenesis and accelerates melanoma metastasis^3^. In contrast, melanoma-bearing B cell-deficient μMT mice fail to develop LN lymphangiogenesis^2^ and show reduced tumor growth^4^. Taken together, these findings suggest that tumors signal to LNs to induce B cell accumulation and inhibit anti-tumor immune responses. Alternatively, tumors could signal via the bloodstream to induce systemic immune responses in the spleen and non-draining LNs, as well as in the TDLN.

B lymphocytes possessing regulatory activity have been identified in mice with cancer and autoimmune diseases^5^,^6^,^7^. These regulatory B cells (Bregs) suppress immune responses independent of their antibody-producing function^7^. Murine Bregs in cancer have previously been studied using melanoma or breast carcinoma cells implanted in the flank^5^,^8^,^9^,^10^. Bregs exert an immunosuppressive effect in autoimmune disease by secreting IL-10 6,7 or by promoting the generation of immunosuppressive regulatory T cells (Tregs) in cancer^8^. Bregs represent a heterogeneous population, and several different subtypes have been identified depending on the particular model studied^11^. B10 (CD1d^hi^CD5^+^), T2-MZP (B220^+^IgM^hi^CD21^hi^CD23^+^), and peritoneal B-1a Bregs can produce IL-10 to suppress autoimmune disease^6^,^7^. Adoptive transfer of tumor-evoked Bregs (tBregs B220^+^CD25^+^) produced *ex-vivo* by culturing B cell with tumor conditioned media produce TGF-β to generate Tregs^8^ which promote metastasis. In the present study, we identify the preferential accumulation of a B cell subset with regulatory activity localized to the TDLN using the B16-F10 melanoma footpad model. These B cells appear to utilize an unconventional mechanism to promote tumor growth.

## Results

### Preferential accumulation of T2-MZP B cells is restricted to the TDLN

**Figure 1:**
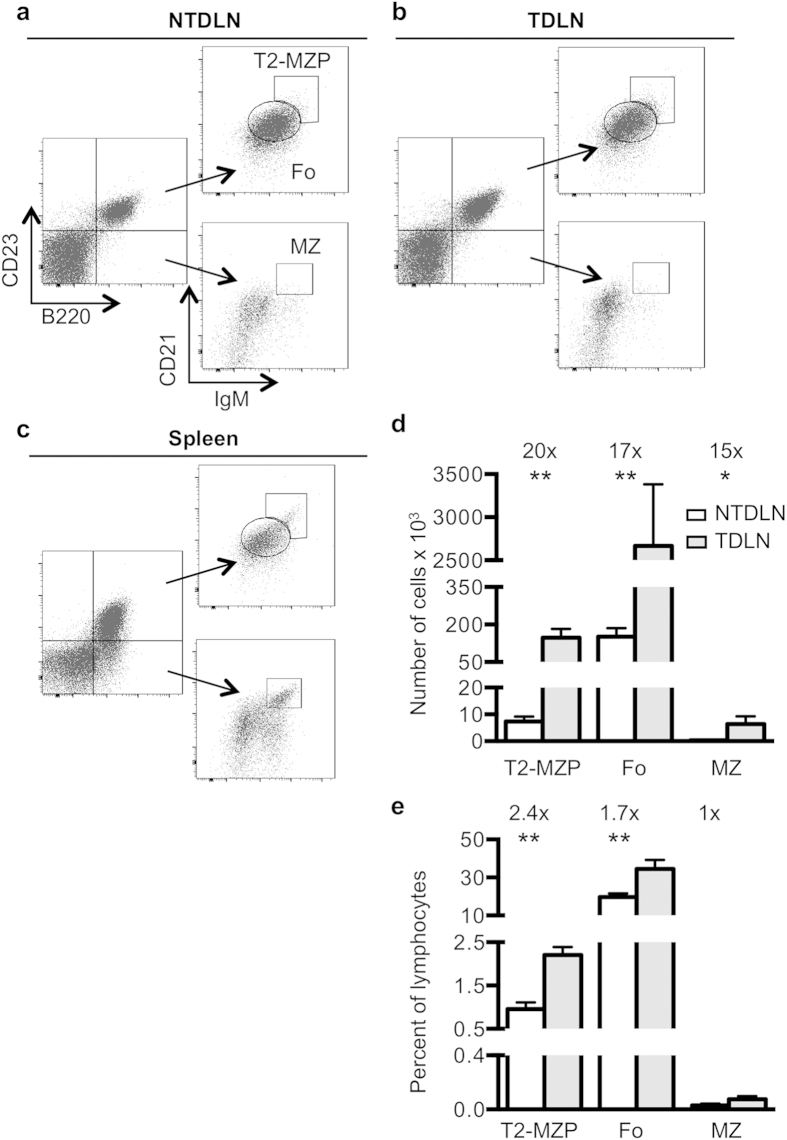
B220^+^CD23^+^ B cells preferentially accumulate in the TDLN. Lymphocytes from tumor-bearing and naïve mice were surface stained and analyzed by flow cytometry. Cells were gated to detect follicular (Fo), T2-MZP, and marginal zone (MZ) B cells in the (**A**) NTDLN (n = 10), (**B**) TDLN (n = 10), and (**C**) spleen (n = 4). (**D**) The absolute number of these three populations and (**E**) percent of lymphocytes are shown. Gating was originally drawn according to the splenic populations and then applied to lymph nodes. Cumulative data from four independent experiments are shown as the mean +/– standard error, with white bars representing the NTDLN populations and light gray bars indicating TDLN populations. Significance was determined using a Wilcoxon signed-rank test for paired samples with *p = 0.0156) and **p = 0.002.

The B16-F10 melanoma rear footpad model allows for the evaluation of tumor-specific alterations in the TDLN, by comparison of tumor-draining versus contralateral non-draining LNs from the same mouse. LN lymphocytes were characterized using surface markers to identify developmentally distinct B cell subsets to test whether TDLN B cell accumulation involves alterations in their phenotype. B cell subsets can be distinguished by B220, IgM, CD23, and CD21 expression. T2-MZP B cells are B220^+^CD23^+^IgM^hi^CD21^hi^, while follicular (Fo) B cells are B220^+^CD23^+^IgM^int^CD21^int^, and marginal zone (MZ) B cells are B220^+^CD23^−^IgM^hi^CD21^hi^ 6. Flow cytometric analysis of these developmental populations in wild-type mice demonstrates that all three B cell populations are present in NTDLNs (Fig. 1a) and TDLNs (Fig. 1b). The MZ B cells represent a small proportion of LN B cells even though they are abundant in the spleen (Fig. 1c). All three subsets are significantly increased in number in TDLNs (Fig. 1d). However, the LN T2-MZP B cell subset shows the greatest preferential accumulation (2.4-fold) with a smaller increase in the frequency of Fo B cells, and no change in the proportion of MZ B cells in the TDLN (Fig. 1e). These findings demonstrate the preferential accumulation of B220^+^CD23^+^ T2-MZP and Fo populations, but not MZ B cells in the TDLN.

### Local accumulation of TDLN B cells

**Figure 2:**
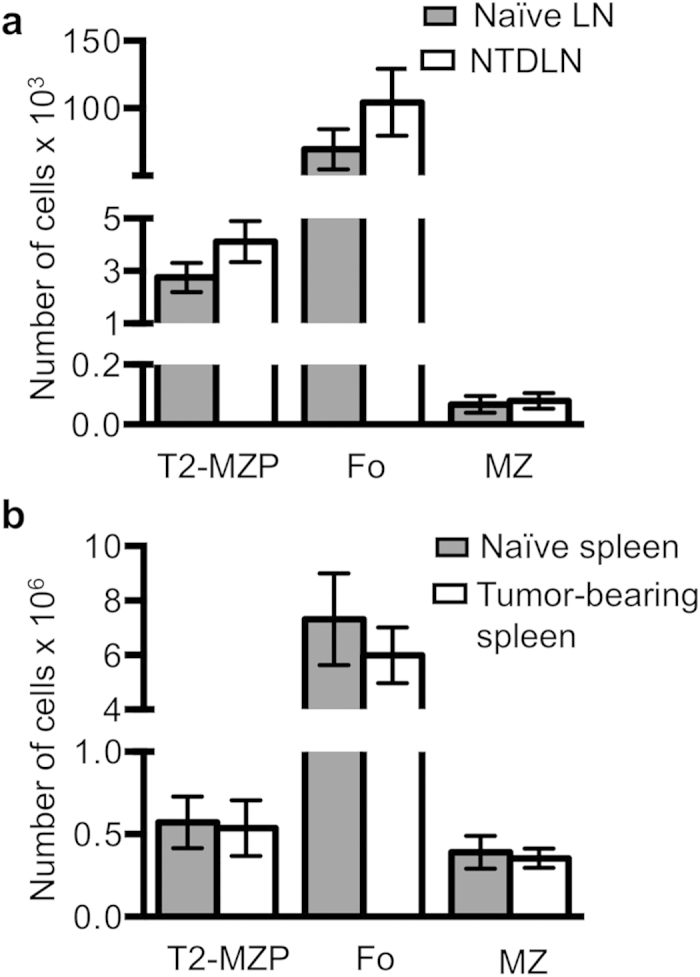
TDLN B cell accumulation is not accompanied by a systemic response. Mice were injected with B16-F10 melanoma cells in the rear footpad (n = 7) and were sacrificed 21 days later. Naïve (tumor-free) littermates were injected with saline (n = 6). (**A**) B cell subsets from popliteal LNs and (**B**) spleens were enumerated by flow cytometry to compare tumor-bearing and naïve mice. Cumulative data from three independent experiments are shown and are depicted as the mean +/– standard error. Naïve mice are represented by dark gray bars, and tumor-bearing mice by white bars. Significance was determined using a Wilcoxon rank-sum test for unpaired samples. No comparisons were significant.

B cell alterations in TDLNs could reflect a local response induced by lymphatic drainage to the LN, or the tumor could exert systemic effects via the bloodstream to induce lymphocyte accumulation in additional secondary lymphoid organs. To determine if lymphoid organs other than the TDLN exhibit B cell alterations in the footpad melanoma model, the uninvolved LNs and spleens from tumor-bearing mice were compared to tumor-free naïve littermates to detect any differences in B cell populations. NTDLNs and naïve popliteal LNs exhibited the same number of the three B cell populations (Fig. 2a) demonstrating that B cell accumulation is restricted to the TDLN, with the NTDLN resembling a naïve LN. Additionally, spleens from tumor-bearing and naïve mice showed similar numbers of each of the three B cell subtypes (Fig. 2b). These findings demonstrate the absence of a significant systemic B cell response to the tumor, and they further indicate that the immune response is localized to the TDLN.

### T2-MZP B cells promote tumor growth

**Figure 3:**
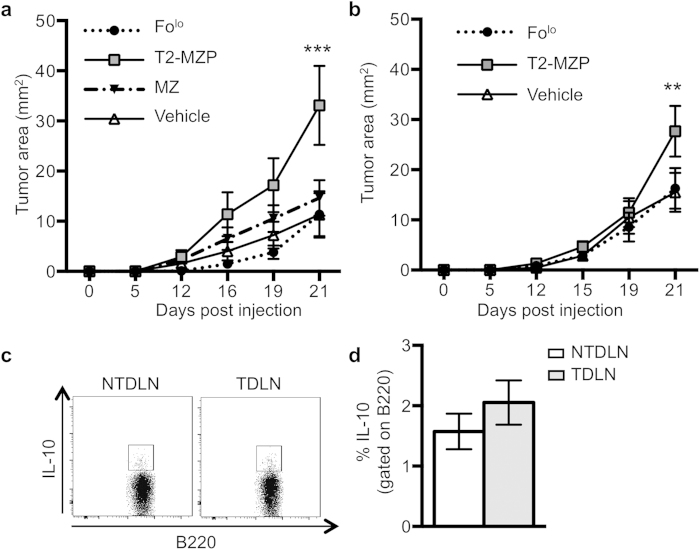
Adoptive transfer of T2-MZP B cells promotes tumor growth. Naïve B cell subsets were FACS-purified and adoptively transferred at the time of tumor implantation. B cell recipients were either (**A**) μMT or (**B**) wild-type mice. Tumor area was calculated by measuring and multiplying perpendicular axes. (**C**) Wild-type mice were sacrificed 21 days after tumor implantation to measure B cell-derived IL-10. PMA/Ionomycin stimulated LN lymphocytes were immunostained and analyzed by flow cytometry to detect B220^+^IL-10^+^ cells. (**D**) The percent of IL-10-producing cells gated on B220^+^ cells is shown. Data are from at least three independent experiments with 6 to 9 mice per group for all experiments. Significance was determined using a two-way repeated measures ANOVA with Bonferroni’s correction for tumor area measurements and a Wilcoxon signed-rank test for paired samples for IL-10 measurements. **p = 0.0005 and ***p < 0.0001 when comparing T2-MZP to saline vehicle control.

T2-MZP B cells have previously been associated with a regulatory function, as transplantation of these cells reduces autoimmune disease by suppressing an anti-host immune response in a mouse model of rheumatoid arthritis^6^. To determine if T2-MZP B cells possess regulatory activity in the footpad melanoma model, B cell-deficient μMT littermates were reconstituted with 1 × 10^6^ naïve splenic FACS-sorted B cell subsets at the time of tumor implantation, and tumor growth was measured over 21 days. LN B cells could not be used for these studies as they are L-selectin^lo^, so that they do not enter LNs efficiently^12^,^13^. Mice receiving T2-MZP B cells grew significantly larger tumors compared to the vehicle control (Fig. 3a) through 21 days. In contrast, mice reconstituted with MZ B cells exhibited no change in tumor growth compared to the saline vehicle control. Reconstitution with either Fo^lo^ B cells, a subset of the Fo population that is well separated from the T2-MZP B cells, or with the entire Fo population also failed to promote tumor growth relative to the vehicle control (Supplementary Figure S1). These results demonstrate that only the T2-MZP B cell subset promotes tumor growth in μMT mice.

Since μMT mice lack peripheral B cells, we sought to address whether adoptive transfer of T2-MZP B cells shows the same activity in wild-type mice with an intact B cell compartment. We hypothesized that the introduction of T2-MZP B cells should also accelerate tumor growth in mice that have a normal immune system. Wild-type littermates were reconstituted with 1 × 10^6^ FACS-sorted naïve B cell subsets (T2-MZP or Fo^lo^) or vehicle at the time of tumor implantation. Mice reconstituted with T2-MZP B cells grew significantly larger tumors at 21 days compared to vehicle control, while the Fo^lo^ transfer again had no effect on tumor growth (Fig. 3b). These findings indicate that transfer of T2-MZP B cells but not other B cell subsets accelerates tumor growth in both μMT and wild-type mice, demonstrating that T2-MZP B cells possess tumor-promoting activity.

Previous studies identified IL-10 as a key mediator of Breg function in autoimmune disease and cancer^11^,^14^, while other studies have found that B cell derived IL-10 is not necessary to mediate immunosuppression^5^,^8^. To determine if B cell-derived IL-10 is increased in the TDLN, LNs from tumor-bearing wild-type mice were collected, stimulated, stained, and analyzed by flow cytometry for intracellular IL-10 (Fig. 3c). There was no change in the frequency of IL-10-producing B cells in the TDLN (Fig. 3d), and this proportion is consistent with what is seen in naïve mice of the same age^15^, suggesting that other mechanisms mediate the tumor-promoting activity of LN B cells.

### TDLN T cell subsets accumulate coordinately

**Figure 4:**
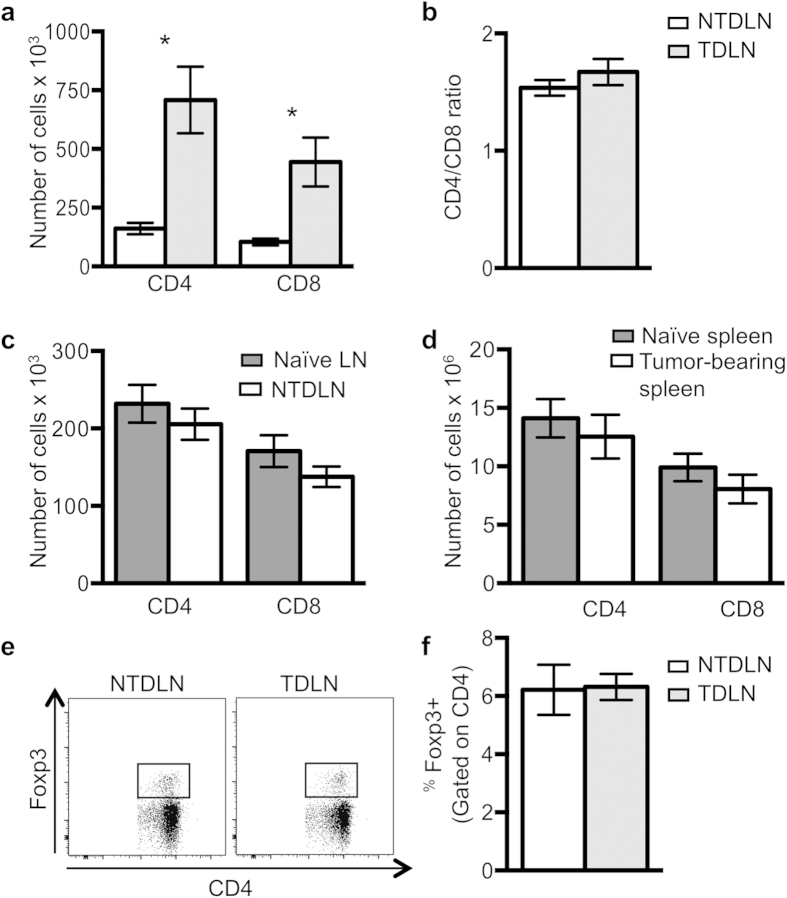
Coordinate accumulation of T cells is restricted to the TDLN. T cells were quantified by flow cytometry from tumor-bearing wild-type mice. (**A**) The absolute number (**B**) and CD4/CD8 ratio are shown (n = 9). Naïve (tumor-free) mice were injected with saline only (n = 6). T cells from (**C**) popliteal lymph nodes or (**D**) spleens were enumerated by flow cytometry. (**E**) The proportion of CD4^+^Foxp3^+^ cells in the LNs (n = 5) was determined by flow cytometry and (**F**) quantified as a percent of CD4^+^ cells. Data are represented as the mean +/– standard error from at least three experiments. Significance was determined using a Wilcoxon rank-sum test for unpaired samples or a Wilcoxon signed-rank test for paired samples with *p = 0.0078.

The TDLN also exhibits increased T lymphocyte content^2^. To determine if CD4 or CD8 T cell subsets differentially accumulate, TDLN and NTDLN T cells were phenotyped by flow cytometry. Both CD4 and CD8 T cells increase 3-fold in the TDLN relative to the NTDLN (Fig. 4a), and the CD4/CD8 ratio remains unchanged in the TDLN (Fig. 4b), demonstrating that both T cell populations coordinately accumulate in the TDLN. T cell accumulation is restricted to the TDLN, as no difference in the number of either population is altered when comparing NTDLNs versus naïve nodes (Fig. 4c), or spleens from tumor-bearing and naïve mice (Fig. 4d). These findings demonstrate that the TDLN is the initial site of the T cell immune response to tumors.

Bregs could potentially exert their regulatory function by promoting the conversion of resting CD4 T cells to CD4^+^Foxp3^+^ Tregs, as been shown with *ex vivo* generated Bregs^8^. LNs from tumor-bearing wild-type mice were analyzed to determine if the TDLN, which features more T2-MZP B cells, contains a greater proportion of Tregs. The flow cytometric profile (Fig. 4e) and frequency of CD4^+^Foxp3^+^ Tregs (Fig. 4f) are the same in the TDLN and NTDLN, indicating that T2-MZP accumulation does not induce Treg generation within the TDLN. The percentages observed in the NTDLN resemble the frequency seen in naïve mice of a similar age^16^. We also examined the effector T (Teff) cell to Treg ratio. A lower ratio in the TDLN compared to the NTDLN would indicate a shift of the anti-tumor immune response since Tregs can inhibit Teff recruitment^17^. No difference in this ratio was measured in the TDLN compared to the NTDLN (Supplemental Figure S2) suggesting that Tregs are not responsible for regulating the immunosuppressive environment within the TDLN.

## Discussion

While Bregs have previously been described in the spleen of mice with cancer or autoimmune diseases^6^,^7^ this is the first demonstration of a Breg population that preferentially accumulates in the TDLN. How these T2-MZP B cells accumulate in the TDLN remains unknown. These cells could arise from proliferation of existing B cells within the TDLN or they could be selectively trafficked to the TDLN from the spleen. Overall, however, B cell accumulation is limited to the TDLN, since no change in B lymphocyte number is detected in the spleen or NTDLN. The modest T cell accumulation is also restricted to the TDLN, while no alterations in particular T cell subsets were noted in either the NTDLN or spleen from tumor-bearing mice compared to naïve mice. These findings support the hypothesis that the early, pre-metastatic, tumor immune response is restricted to the TDLN. The absence of a systemic B or T lymphocyte response to these small (10–35 mm^2^) tumors further indicates that immune alterations detected within the TDLN are the result of local signaling between the tumor and the draining lymph node. In this model, the immune response to the tumor is likely mediated by soluble material from the tumor or antigen-presenting cells entering the LN via the lymphatic drainage from the tumor to the popliteal LN^18^. These findings demonstrate that TDLNs are a specialized environment where the immune response to tumors first develops. Other studies of tumor immune responses in mouse flank models have identified systemic immune responses in the spleen and NTDLNs that were not detected in our footpad tumor model^9^,^10^,^19^,^20^. Indeed, the type of immune response generated could depend on factors such as tumor size, the anatomical location of tumor implantation, or the type of cancer involved^21^.

A single introduction of naïve T2-MZP B cells, but not other B cell subsets, accelerates tumor growth in both μMT and wild-type mice. We previously showed that B cells are not detectible in footpad tumors of wild-type mice^2^, suggesting that B cells act within the TDLN rather than in the tumor microenvironment. While the mechanism by which the introduction of such a small number of Bregs accelerates tumor growth remains to be determined, it likely involves amplification of the initiating B cell-derived signal. Further investigation is required to determine how the early TDLN immune alterations relate to the anti-tumor immune responses.

Our finding that TDLN B cells do not produce increased IL-10 is consistent with reports that Breg activity does not necessarily involve IL-10 5,8. While the percent of T2-MZP B cells increases almost 3-fold in the TDLN, the proportion of IL-10-producing B cells does not change, suggesting that IL-10 expression is not restricted to T2-MZP B cells. This further supports the conclusion that IL-10 is likely not the regulatory mechanism utilized by this subset. Instead, TDLN T2-MZP B cells could exert an immunosuppressive effect by inducing LN lymphangiogenesis, as TDLNs from wild-type mice demonstrate extensive LN lymphangiogenesis not seen in the NTDLN^2^ Furthermore, lymphangiogenesis has been shown to be a B cell-dependent process. TDLNs from μMT mice lacking B cells do not demonstrate the same degree of lymphatic sinus growth as their wild-type counterparts^2^. Other studies have provided evidence for a role for lymph node lymphatic endothelial cells in mediating tolerance by deleting antigen-specific T cells^22^,^23^. Alternatively, B cells could prevent an anti-tumor immune response by producing antibodies. For example, low affinity antibodies binding to Fc receptors can result in the recruitment of pro-tumorigenic mast cells and macrophages to tumors^24^. However, the small number of naïve Bregs introduced in our studies would seem insufficient to generate much tumor-specific antibody during the 21-day interval tested. Finally, B cells can polarize macrophages to a type-2 state^25^, which could promote tumor growth^24^. The footpad melanoma model will be useful to investigate the mechanism by which TDLN B cells promote tumor growth, and whether this involves immune activation or suppression within the tumor microenvironment.

Taken together, these studies identify pre-metastatic, local immune responses to the tumor, which include the preferential accumulation of a TDLN-resident Breg population that could generate an immunosuppressed environment within the TDLN. Similar alterations could be involved in human cancer, as TDLNs from patients with various types of solid tumors also significantly accumulate B cells^26^,^27^. An understanding of the mechanism by which TDLN T2-MZP Bregs promote tumor growth could inform the development of therapeutic strategies to permit a robust anti-tumor immune response.

## Materials and Methods

### Mouse Melanoma Model

C57BL/6J and μMT mice from Jackson Laboratory (Bar Harbor, ME) were housed in a specific pathogen-free facility. Animals were handled according to institutional guidelines. Five week-old male and female littermates were injected subcutaneously with B16-F10 melanoma in one hind footpad, and saline was introduced in the contralateral footpad^2^. B16-F10 cells were obtained from ATCC (ATCC® CRL6475^TM^; Manassas, VA). Early passage cells were tested for viral contamination by RADIL PCR (University of Missouri, Columbia, Missouri) and mycoplasma contamination by MycoAlert^TM^ Mycoplasma Detection Kit (Lonza, Walkersville, MD) prior to injections. For tumor area measurements, perpendicular axes were measured and multiplied. All measurements were conducted while blinded to the experimental condition. Experimental methods were carried out in accordance with approved guidelines by the FHCRC and University of Washington Animal Care and Use Committees.

### Flow Cytometry Analysis

The following anti-mouse antibodies were purchased from BioLegend (San Diego, CA) unless otherwise specified: purified anti-CD16/32 (Tonbo, San Diego, CA), B220, CD23, IgM (eBiosciences, San Diego, CA), CD21/35, CD4, CD8, IL-10, and Foxp3.

Lymphocytes from spleen and lymph nodes of C57BL/6J mice were harvested^2^. Cells were incubated with purified anti-CD16/32 and surface stained then fixed. Intracellular staining was carried out using the BD Cyofix/Cytoperm^TM^ kit following manufacturer’s protocol. For IL-10 analysis, lymphocytes were harvested, stimulated for 5 hours with 50 ng/ml PMA and 500 ng/ml Ionomycin with GolgiStop (contains monensin) (BD, San Jose, CA) and stained^5^. Analysis was conducting using a BD LSRII or BD CantoII flow cytometer and FlowJo software (Tree Star Ashland, OR).

### Adoptive Transfer of B Cell Subsets

Spleens harvested from 4–6 week old C57BL/6J mice were pooled, homogenized, and filtered through a 70 μm cell strainer. Single-cell suspensions were depleted of erythrocytes, surface stained, and sorted into follicular (Fo), follicular low (Fo^lo^), Transitional 2-marginal zone precursor (T2-MZP), and marginal zone (MZ) B cell subsets by a BD FACSAriaIII using gates drawn according to previous reports^6^. B cell subsets at 1 × 10^6^ cells per 100 μl in Hanks Balanced Salt Solution (HBSS) were transferred retro-orbitally into 4–5 week old μMT or wild-type mice at the time of tumor implantation.

### Statistics

All experiments were repeated a minimum of three times with at least two mice per cohort. Statistical analyses were performed using GraphPad Prism 6 and were carried out using a Mann-Whitney *U* test for unpaired samples, a Wilcoxon signed-rank test for paired samples, or a two-way repeated measures ANOVA with Bonferroni’s correction for tumor area measurements. Normality was assessed using the Anderson Darling test, and a two-tailed analysis was carried out for all comparisons.

## Additional Information

**How to cite this article**: Ganti, S. N. *et al.* Regulatory B cells preferentially accumulate in tumor-draining lymph nodes and promote tumor growth. *Sci. Rep.* **5**, 12255; doi: 10.1038/srep12255 (2015).

## Supplementary Material

Supplementary Information
