# Supplementary material for: Regulatory B cells preferentially accumulate in tumor-draining lymph nodes and promote tumor growth
Source: Sci Rep. 2015 Jul 20;5:12255. doi: 10.1038/srep12255 (PMC4507466; doi:10.1038/srep12255)
Supplement: Supplementary Information [file srep12255-s1.doc]

**Title:** Regulatory B cells preferentially accumulate in tumor-draining lymph nodes and promote tumor growth

**Authors**: Sheila N. Ganti^1^, Tina C. Albershardt^2^ Brian M. Iritani^1^, Alanna Ruddell* ^1, 2^

1 Department of Comparative Medicine, University of Washington, Seattle, WA, USA

2 Fred Hutchinson Cancer Research Center, Seattle, WA, USA

**Supplemental Figure 1: Fo and Fo^lo^ transfer result in similar tumor size**

(A) 1 x 10^6^ Fo or Fo^lo^ B cells, isolated using the diagrammed gating scheme, were transferred to μMT mice at the time of tumor implantation. (B) Tumor area was measured regularly with at least six mice/group and three independent experiments. No significant differences were observed at any time point as determined by a two-way repeated measures ANOVA with Bonferroni’s correction.


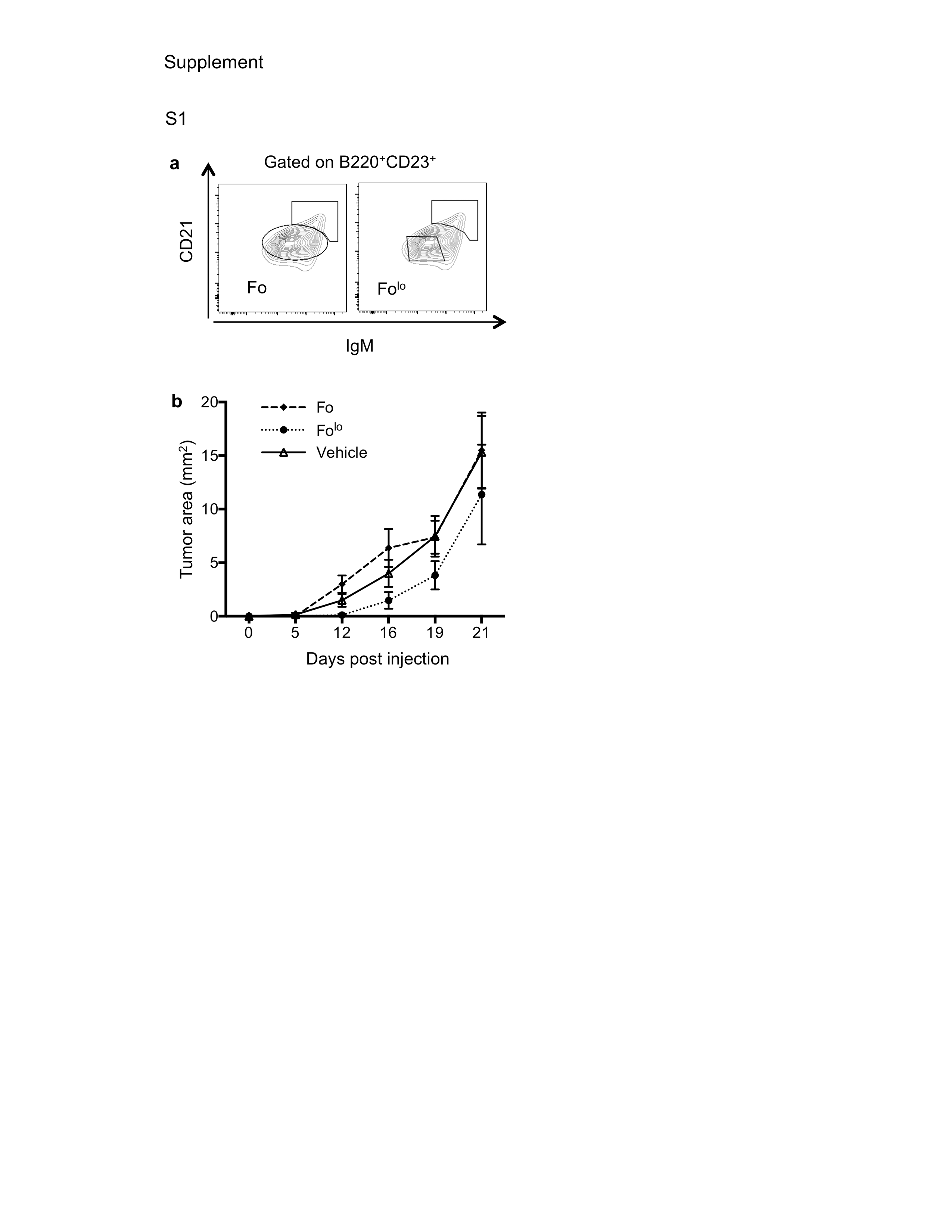


**Supplemental Figure 2: The Teff/Treg ratio is unchanged in the TDLN**

The number of CD4, CD8, and Treg cells were determined for both the NTDLN and TDLN based on flow cytometric data. The CD4/Treg and CD8/Treg ratios were calculated for each sample. Cumulative data from two independent experiments are shown and are depicted as the mean +/- standard error. NTDLNs are represented by white bars and TDLNs by gray bars. No significant differences were observed as determined by a Wilcoxon signed-rank test for paired samples (n=5).

Teff/Treg

NTDLN

TDLN

CD4

CD8
